# Gender differences in criminal justice system involvement and associations with post-deployment factors among Post-9/11 veterans

**DOI:** 10.1186/s40352-026-00432-1

**Published:** 2026-06-24

**Authors:** Julia Naganuma-Carreras, Elizabeth Collazo, Nicholas Livingston, Jaclyn Kearns, Julia Sager, Daniel Lee, Terence Keane, Brian Marx

**Affiliations:** 1 VA Boston Healthcare System, Boston, USA; 2 Boston University, Boston, USA

**Keywords:** Veterans, Criminal justice system, Gender differences, Mental health, Alcohol use disorder, Homelessness

## Abstract

**Background:**

Veterans are overrepresented in the United States criminal justice system, yet limited research has examined how mental health, substance use, and psychosocial factors intersect to shape involvement in the justice system—particularly across gender. As the population of women veterans grows, understanding gender-based differences in criminal justice involvement is critical to developing targeted, patient-centered interventions.

**Methods:**

We analyzed data from 1,360 post-9/11 veterans (*M*_*age*_ = 37.49; 51.3% women) to examine gender-specific associations between individual and co-occurring factors and criminal justice involvement. Justice involvement was defined as being arrested and charged with a crime after deployment, which was measured through self-report.

**Results:**

Overall, 15.0% of veterans reported post-deployment justice involvement (11.3% of women and 18.7% of men). Several factors were related to higher likelihood of post-deployment justice involvement in both groups (i.e., younger age, provisional alcohol use disorder diagnosis) while others were only related in one group (e.g., problematic anger among women; history of homelessness among men).

**Conclusions:**

Our findings underscore the importance of integrated, gender-responsive approaches to reduce justice involvement among veterans. Public health and criminal justice system interventions should address overlapping domains of need, including mental health, housing instability, and substance use, while tailoring strategies to gender-specific risk profiles.

Involvement in the criminal justice system is considerably higher among veterans than non‑veterans. Nationally representative surveys indicate that almost one‑third of veterans (31.1%) have a lifetime history of arrest, compared to 18% of non‑veterans (Snowden et al., 2017). Each year, between 100,000 and 180,000 veterans are incarcerated, representing approximately 8% of the total incarcerated population in the United States (Bronson et al., 2015; Maruschak et al., 2021). Justice‑involved veterans frequently experience multiple, overlapping vulnerabilities, including elevated rates of trauma exposure, mental health conditions, substance use disorders, and homelessness (Blodgett et al., 2015; Edwards et al., 2023). The convergence of criminal justice involvement with these overlapping challenges creates substantial barriers to post‑deployment reintegration into civilian life, including recovery from mental and physical injuries, housing stability, and readjustment to social roles. Given both their number and complexity of need, veterans involved with the criminal justice system are considered a high‑priority subgroup within the Department of Veterans Affairs healthcare system (VA OMHSP 2024).

The transition from military to civilian life constitutes a critical context in which veterans face elevated risk for clinical, behavioral, and social challenges that may increase vulnerability to justice involvement (Derefinko et al., 2019; Zogas 2017). Following military deployment, many service members enter a vulnerable transition period characterized by limited support services, including inadequate screening for severe psychological distress or problematic substance use, heightened social isolation (e.g., loss of friendships and peer networks, readjusting to family separation), and socioeconomic and educational challenges (e.g., lack of employment plans or stable housing, increasing risk for poverty and homelessness; Derefinko et al., 2019). In addition, prior research has documented a high prevalence of problematic anger among recently deployed veterans and its associations with posttraumatic stress disorder (PTSD), relationship difficulties, and economic stressors (Adler et al., 2022), as well as suicidality and violence (Varker et al., 2022). Beyond their individual effects, these risk factors frequently co‑occur, exacerbating mental health and substance use problems, contributing to functional difficulties, and heightening emotional dysregulation, which may in turn increase the likelihood of justice involvement (Backhaus et al., 2016; Edwards et al.,^2025^; Elbogen et al., 2012). Consistent with these challenges, veterans experience elevated rates of criminal justice involvement relative to the general population (Snowden et al., 2017).

Elevated rates of criminal justice involvement among veterans are thought to reflect, in part, the high burden of mental health, substance use, and social challenges in this population (Blodgett et al., 2015). A recent meta‑analysis of over 1.2 million veterans with criminal‑legal involvement estimated that approximately 8 in 10 experience at least one mental health condition, 7 in 10 have a substance use disorder, 1 in 10 have a history of suicidal ideation, and 4 in 10 have a history of homelessness (Edwards et al., 2025). Notably, these factors, including substance use disorders (Tsai et al., 2014), PTSD (Carlson et al., 2013; Taylor et al., 2020), lifetime history of suicide attempt (Edwards et al., 2025), and homelessness (Kelton et al., 2025), have been repeatedly linked to elevated risk of lifetime involvement in the justice system. Additionally, other service‑related factors, such as combat exposure and traumatic brain injury, have also been associated with criminal justice involvement among veterans (Blodgett et al., 2015; Carlson et al., 2013; Legal Services Corporation 2022; Taylor et al., 2020).

Although these clinical and social vulnerabilities are widely documented among veterans with justice involvement, emerging evidence suggests these risk factors may not operate uniformly and instead vary by gender. Specifically among justice‑involved veterans, women consistently present with higher rates of mental health diagnoses (e.g., depression, anxiety; Desai et al., 2023; Finlay et al., 2015), whereas men more often present with alcohol and substance use disorders (Finlay et al., 2015; McCall & Tsai 2017). Extant literature also suggests that justice‑involved women veterans are more likely than men to report a history of homelessness (Desai et al., 2023) and that homeless female veterans may report greater unmet needs (Tsai et al., 2023). Evidence on gender differences in PTSD among justice‑involved veterans indicates that PTSD is generally more prevalent among women (Finlay et al., 2015; Stainbrook et al., 2016), although combat‑related PTSD may be more common among men (McCall & Tsai 2017). Despite this growing body of research on gender differences, most studies among veterans have relied on predominantly male samples and have treated gender primarily as a covariate rather than explicitly examining gender‑specific associations. Consequently, research on veteran women remains limited, and their specific risks and needs related to criminal justice involvement are poorly understood. Studies that meaningfully include and center veteran women are urgently needed to better characterize clinical, social, and trauma‑related needs in relation to justice involvement and to inform more effective, gender‑responsive prevention and intervention approaches.

The need for gender-responsive and patient-centered prevention and treatment strategies is especially concerning in light of the broader consequences of criminal justice system contact. Justice involvement is not only an outcome in itself; it can also trigger a cascade of adverse consequences, including job loss, housing instability, family disruption, worsening mental health and substance use, and increased risk of self‑harm (Derefinko et al., 2019; Holliday et al., 2021). These risks may be especially pronounced in veterans during the post‑deployment period, underscoring the importance of early intervention following deployment. Identifying multi‑domain risk profiles associated with justice involvement can help clinicians and systems intervene earlier, ideally before arrest or incarceration occurs, by targeting combinations of risk factors that place veterans on trajectories toward the criminal justice system. Such evidence is critical to inform the design of post‑deployment treatment and recovery services, as well as diversion and reintegration programs, particularly for women veterans who have been largely overlooked in prior work. As such, the present study draws on a large, gender-balanced cohort of 1,360 post-9/11 United States veterans assessed after their return from deployment to Iraq and/or Afghanistan. Our primary aim was to examine gender differences in the associations between a broad set of demographic, clinical, and deployment-related factors and post‑deployment criminal justice involvement. To address this aim, we examined these associations separately for women and men and compared patterns of association across genders to identify both shared and gender-specific correlates of criminal justice involvement. By characterizing gender‑specific risk profiles, this study seeks to inform more patient‑centered, holistic, and gender‑responsive prevention and intervention strategies for veterans at risk of criminal justice involvement.

## Method

### Participants and procedure

Participants were 1,360 United States veterans enrolled in the Veterans After-discharge Longitudinal Registry (Project VALOR) who deployed at least once to Iraq and/or Afghanistan and subsequently enrolled in Veterans Health Administration services. During recruitment, we oversampled for veterans with PTSD and veteran women; those with a chart diagnosis of PTSD were recruited on a 3:1 ratio and veteran women on a 1:1 ratio to ensure adequate representation of individuals with PTSD and veteran women (see Rosen et al., 2012; Wisco et al., 2014 for greater details regarding study design and recruitment). For this study, we focused on the second assessment period (T2) as this wave was the first to collect information regarding involvement in the criminal justice system. Participants completed T2 assessments at a mean of 2.47 years (*SD* = 0.52) following the first assessment period, which occurred in approximately 2009. Of the full sample, 1,367 (82.90%) completed at least one measure at T2. We present descriptive characteristics of the sample in Table 1.

**Table 1:** Demographic and clinical characteristics of full sample

Demographics	***M* (*SD*)**
Age	40.66 (9.80)
	*n* (%)
Sex (Male)	824 (50.0)
Race (Non-Hispanic White)	917 (67.3)
Marital Status (Married)	720 (52.5)
Sexual Orientation (Heterosexual)	1265 (92.3)
Current diagnoses	
Alcohol use disorder	358 (26.2)
Generalized anxiety disorder	382 (28.3)
Major depressive disorder	491 (36.3)
Somatoform disorder	543 (40.4)
Posttraumatic stress disorder	846 (63.0)
Past-month suicidal ideation	498 (37.2)
Lifetime history of suicide attempt	326 (24.2)
History of post-deployment homelessness	194 (14.2)
Lifetime history of TBI	924 (56.0)

Participation involved a telephone interview and online questionnaire battery. Interviewers were doctoral-level psychologists who were fully trained in all included interview measures. All participants provided informed consent before beginning any study procedures. All study procedures were approved by the (omitted for masked review) Institutional Review Board and the Human Research Protection Office of the United States Army Medical Research and Materiel Command.

### Measures

We assessed demographic characteristics (gender, age, race/ethnicity, education, marital status, military branch, and number of deployments) via self‑report questionnaire. Post-deployment criminal justice involvement was assessed with a single item: “Were you arrested and charged with a crime after your first Operation Enduring Freedom/Operation Iraqi Freedom/Operation New Dawn deployment?”. Additionally, we assessed post-deployment history of homelessness with a single item: “Since your first deployment, did you ever experience a period of homelessness?”. Clinical constructs included current provisional diagnoses of PTSD and alcohol use disorder, lifetime traumatic brain injury, past‑month suicidal ideation and lifetime suicide attempt, as well as provisional diagnoses of major depressive disorder, generalized anxiety disorder, and somatoform disorder. Additional risk and resilience factors included alcohol use, combat exposure, aftermath of battle, and post‑deployment social support, functional impairment, life stressors/trauma, and anger.

Measures and their corresponding psychometric properties are summarized in Table 2. For multi-item self-report scales, internal consistency estimates (Cronbach’s α) were calculated in the current sample. For structured clinical interviews and algorithm-derived variables (e.g., SCID-5 PTSD diagnosis, MINI suicidality module, and TBI variables), internal consistency estimates were not computed, as these measures are not unidimensional scales but instead rely on diagnostic or rule-based scoring procedures. For these measures, prior evidence of reliability and validity is provided via cited sources, including studies conducted in military and veteran samples.

**Table 2:** Summary of measures

Construct	Measure	Reliability Statistic	Project VALOR administration	References
PTSD	Structured Clinical Interview for *DSM-5* PTSD module	N/A	Telephone interview	First et al., 2015; Green et al., 2017
Traumatic brain injury (TBI)	Defense and Veterans Brain Injury Center TBI questionnaire, modified for brief administration	N/A	Telephone interview	Brown et al., 2005; Levin, 1995; Jackson et al., 2016; Wilde et al., 2006
Mental health disorders (somatoform disorder, depression, anxiety)	Prime-MD Patient Health Questionnaire (PHQ)	α = .81, .90, and .85, respectively	Self-administered questionnaire	Eakman et al., 2019, Spitzer et al., 1999, Wells et al., 2013
Alcohol use	Alcohol Use Disorder Identification Test (AUDIT)	α = .88	Self-administered questionnaire	Reid et al., 1999; Saunders et al., 1993
Combat-related experiences	Deployment Risk and Resilience Inventory (DRRI) Section I (Combat Experiences) and Section J (Post-Battle Experiences)	α = .91 and .92, respectively	Self-administered questionnaire	King et al., 2006
Social support	DRRI Section L (Postdeployment support)	α = .84	Self-administered questionnaire	King et al., 2006
Functional impairment	Inventory of Psychosocial Functioning (IPF)	α = .95	Self-administered questionnaire	Bovin et al., 2018; Klein et al., 2024, Marx et al., 2020
Life stressors/trauma	Life Events Checklist (LEC)	α = .73	Self-administered questionnaire	Gray & Lombardo, 2004
Anger	Dimensions of Anger Reactions, revised short form (DAR-5)	α = .90	Self-administered questionnaire	Forbes et al., 2004; Hawthorne et al., 2006; Novaco et al., 2012
Suicidal ideation	Mini-International Neuropsychiatric Interview (MINI) suicide module	N/A	Telephone interview	Klein et al., 2024; Lecrubrier et al., 1997; Sheehan et al., 1998; Tsai et al., 2018
Suicide attempt	Single item: “Have you ever made an actual attempt to kill yourself in which you had at least some intent to die?” from Self-Injurious Thoughts and Behaviors Interview (SITBI)	N/A	Telephone interview	Nock et al., 2007; Stanley et al., 2023

### Data analyses

All analyses were conducted using IBM SPSS Statistics (Version 29, IBM Corporation). The primary outcome was post-deployment criminal justice system involvement, coded as a binary variable (0 = no arrest/charges post-deployment, 1 = arrested/charged with a crime post-deployment). Multivariable logistic regression models were used to examine factors associated with post-deployment criminal justice involvement separately for male and female veterans. We calculated odds ratios and 95% confidence intervals to estimate the magnitude of association between each binary and continuous variable and post-deployment criminal justice involvement. Continuous variables were entered as such to preserve information and statistical power, whereas categorical variables were included using indicator coding. All predictors were entered simultaneously into each model to estimate the independent association between each variable and criminal justice involvement, while accounting for the potential influence of other relevant demographic, clinical, and deployment-related variables. Prior to conducting regression analyses, patterns of missing data were examined using descriptive statistics to evaluate the extent of missingness across study variables and whether missingness was associated with demographic and clinical variables. Of the sample included in analyses, most participants (71.4%) were not missing any included variables; an additional 8.89% were only missing one variable. Analyses examining patterns of missing data indicated that missingness was not significantly associated with most study variables. However, participants who screened positive for major depressive disorder and those reporting higher levels of combat exposure were somewhat more likely to have missing predictor data. As these variables were included in the models, we consider model inferences based on data missing at random (MAR). Multicollinearity among predictors was assessed by examining variance inflation factors and tolerance statistics for the sex-straified models. All variance inflation factor values were below acceptable thresholds (< 5) and tolerance values exceeded recommended minimum levels (> .20), indicating that multicollinearity was not a concern.

## Results

Among participants included in analyses, 204 (15.0%) of respondents indicated they had been arrested and charged with a crime following return from deployment. Overall, 11.3% of veteran women (*n* = 79) and 18.9% of veteran men (*n* = 125) reported a history of post-deployment criminal justice involvement (χ^2^ = 15.07, *df* = 1, *p* < .001).

### Shared correlates across both genders

We present results from sex-stratified multivariable logistic regression analyses identifying factors associated with post-deployment criminal justice involvement in Table 3. Age and alcohol use disorder were significantly associated with involvement in the criminal justice system among both veteran women and men. Specifically, each one year increase in age was associated with lower odds of criminal justice system involvement for women (OR = 0.951, *p* = .024) and men (OR = .957, *p* = .006). In contrast, screening positive for alcohol use disorder was associated with significantly higher odds of criminal justice involvement for both genders. Veteran women who screened positive for alcohol use disorder had 3.49 times higher odds of criminal justice involvement (*p* < .001), whereas veteran men who screened positive for alcohol use disorder had 2.85 times higher odds of criminal justice system involvement (*p* < .001).

**Table 3:** Multivariate logistic regression model of post-deployment CJS involvement among female and male veterans

Factor	Female				Male			
		95% CI				95% CI		
	Odds ratio	LL	UL	*p*	Odds ratio	LL	UL	*p*
Demographics								
Age	**.951**	**.910**	**.993**	**.024**	**.957**	**.927**	**.988**	**.006**
Race (Reference: White)								
Hispanic/Latinx	–	–	–	–	.199	.022	1.80	.151
American Indian or Alaskan Native	–	–	–	–	1.70	.140	20.7	.677
Asian	–	–	–	–	.951	.077	11.7	.969
Native Hawaiian or Other Pacific Islander	–	–	–	–	–	–	–	–
Black	.830	.335	1.00	.687	.845	.310	2.30	.741
Multiracial	1.77	.783	.687	.170	.951	.424	2.13	.904
Provisional diagnoses								
Alcohol Use Disorder	**3.49**	**1.78**	**.170**	** < .001**	**2.85**	**1.62**	**5.01**	** < .001**
Somatoform Disorder	.869	.419	< .001	.705	**.400**	**.203**	**.788**	**.008**
Major Depressive Disorder	1.14	.468	.705	.771	1.38	.673	2.84	.378
Generalized Anxiety Disorder	1.50	.651	.771	.341	1.19	.586	2.45	.622
PTSD	.899	.396	.341	.798	.922	.466	1.82	.816
Deployment Factors								
Combat exposure	.959	.918	.798	.066	1.01	.979	1.04	.543
Aftermath of battle	1.02	.993	.066	.123	1.01	.980	1.04	.529
Post-deployment social support	.993	.963	.123	.661	.982	.955	1.01	.198
TBI								
Lifetime history of TBI	–	–	–	–	.702	.185	2.66	.602
Number of TBIs	.876	.542	1.00	.588	1.03	.809	1.31	.807
TBI Severity (Reference: None)								
Mild	–	–	–	–	2.48	.808	7.62	.112
Moderate-Severe	–	–	–	–	2.39	.666	8.57	.182
Past-month suicidal ideation	1.46	.511	1.00	.479	.890	.410	1.93	.768
Lifetime history of suicide attempt	2.48	.891	.479	.082	**2.76**	**1.15**	**6.61**	**.023**
History of post-deployment homelessness	1.98	.918	.082	.082	**3.67**	**1.84**	**7.32**	** < .001**
Cumulative trauma	1.05	.940	.082	.363	.982	.900	1.07	.677
Functional impairment	1.00	.972	.363	.984	1.00	.977	1.03	.964
Problematic anger	**1.09**	**1.00**	**.984**	**.029**	.981	.919	1.05	.554

Values in bold are significant at *p* < 0.05

Estimates were not reported for categories with sparse data (n < 10, Odds ratios > 999) resulting in unstable model estimates

### Gender-specific correlates

Among veteran women only, higher levels of problematic anger were significantly associated with criminal justice system involvement. Specifically, each one-point increase in problematic anger severity was associated with a 9% greater odds of criminal justice involvement among veteran women (OR = 1.09, *p* = .029). Among veteran men only, somatoform disorder, lifetime history of suicide attempt, and history of post-deployment homeless were significantly associated with criminal justic system involvement. Men who screened positive for somatoform disorder had lower odds of criminal justice involvement (OR = .400, *p* = .008). In contrast, veteran men with a lifetime history of suicide attempt had 2.76 times higher odds of criminal justice involvement (*p* = .023), and those with a history of post-deployment homelessness had 3.67 times higher odds of criminal justic involvement (*p* < .001), compared to veteran men without these histories. Of note, involvement in the justice system did not significantly differ on the basis of race or ethnicity for either gender.

## Discussion

We examined the prevalence of post‑deployment criminal justice involvement using a nationally representative, gender‑balanced sample of combat-deployed post-9/11 veterans and compared factors associated with involvement between veteran women and men. Overall, 15% of veterans reported a history of post‑deployment criminal justice involvement, including 11.3% of women and 18.9% of men. Younger age and provisional diagnoses of alcohol use disorder were associated with increased likelihood of justice system involvement across veteran women and men. Nevertheless, important gender differences emerged, such that across all examined variables, veteran women and men exhibited distinct, gender‑specific risk profiles for criminal justice involvement.

Although men remain the overwhelming majority of the veteran population, women represent a rapidly growing demographic group served in Veterans Health Administration (VA Office of Women’s Health 2024) and are projected to comprise 18% of both military and veteran populations by 2040 (Sussman 2021). The proportion of women veterans involved in the justice system is also increasing; the incarcerated women veteran population grew by 15% between 2008 and 2018 (Hartley & Baldwin 2024). In our sample, problematic anger emerged as a salient correlate of criminal justice involvement uniquely among veteran women. Prior research suggests that problematic anger may be particularly pronounced in this group, with studies documenting the persistence of heightened irritability and anger at posttreatment among veteran women who have completed PTSD treatment (Larsen et al., 2019; Schnurr & Lunney 2019). These risks may be compounded by evidence that women’s anger expression tends to elicit greater social and institutional sanctions (Carrére et al., 2005; Kaway et al., 2021; Van Doren & Soto 2021). Taken together, such findings may be particularly relevant to veteran women recently returning from military deployment, as anger outbursts have been linked to readjustment difficulties and delayed mental health treatment seeking (Derefinko et al., 2019). Despite evidence that women veterans experience similar levels of anger as their male counterparts (Morland et al., 2020), problematic anger remains under‑researched among women, and this pattern among women warrants further investigation.

In contrast, among veteran men only, history of suicide attempt, past homelessness, and a provisional diagnosis of somatoform disorder were associated with criminal justice involvement. Several of these results support previous findings wherein history of suicide attempt (Edwards et al., 2025) and history of homelessness (Kelton et al., 2025) have been linked to criminal justice involvement. These results underscore the importance of assessing for and addressing suicide risk and homeslessness among veterans post-deployment, especially those with histories of, or as potential risk factors for, criminal justice involvement. To this end, the Veterans Health Administration has implemented universal screening for suicide risk (Gujral et al., 2023) and homelessness (Montgomery 2021) across VA hospitals nationally. However, a unique finding in this study was the association between a provisional diagnosis of somatoform disorder and lower odds of criminal justice involvement among veteran men. One possible explanation for this association is that individuals with somatoform disorders tend to present with functional impairments (Puri & Dimsdale 2011) that may discourage or limit engagement in behaviors that lead to criminal justice involvement. Additionally, individuals presenting with somatoform disorders are more likely to utilize healthcare services (Puri & Dimsdale 2011), increasing the likelihood that these individuals may be connected to supportive services earlier than others, especially considering universal screenings implemented across the Veterans Health Administration nationally. However, given that this study relied on a provisional screening measure rather than a confirmed clinical diagnosis, this finding should be interpreted cautiously.

Notwithstanding differences between veteran women and men, younger age and provisional diagnosis of alcohol use disorder were associated with higher odds of post-deployment criminal justice involvement across both veteran women and men. These findings are consistent with prior research indicating that younger age (Kelton et al., 2022) and alcohol use disorder (Edwards et al., 2025) are associated with criminal justice involvement among veterans. Importantly, although some prior work has found that justice-involved veteran women demonstrate lower odds of alcohol use disorder relative to men (Finlay et al., 2015), our findings highlight that alcohol use remains an important correlate of criminal justice involvement across genders. These results underscore the importance of screening for and addressing problematic alcohol use among veterans, particularly younger veterans, as part of prevention and early intervention efforts aimed at reducing risk for criminal justice involvement.

### Limitations

Although the present study has several strengths, important limitations should be considered when interpreting the findings. First, criminal justice involvement was assessed using a single self-report item (“Were you arrested after service?”), which limited our ability to capture the frequency, type, and severity of justice involvement. As a result, potentially meaningful distinctions among different forms of criminal justice contact (e.g., arrest, incarceration) could not be examined. Reliance on self-report may also introduce bias related to stigma or social desirability, and patterns of reporting could potentially differ by gender. Second, small sample sizes among minoritized racial/ethnic groups limited statistical power to detect associations with race and ethnicity. Although we did not observe significant associations between race/ethnicity and criminal justice involvement in the present analyses, this contrasts with prior research documenting elevated rates of justice involvement among minoritized racial/ethnic groups, particularly Black veterans (Kelton et al., 2022; Tsai et al., 2022). Additionally, our analyses were also restricted to binary gender categories, precluding examination of gender diversity. Emerging research suggests that disparities in both criminal justice involvement and access to care are pronounced among sexual and/or gender minorities. Finally, the cross-sectional study design precludes conclusions about the directionality or causal nature of the observed associations.

### Implications and future directions

The findings of the current study have several important clinical, social, and policy implications for supporting veterans during the transition from military to civilian life and for reducing criminal justice involvement. First, our findings further support the connection between alcohol use, history of suicide attempts, and history of homelessness among justice-involved veterans. These results underscore the important of continued universal screening for suicide risk and homelessness within the Veterans Health Administration to facilitate early identification and connection to appropriate resources (Gujral et al., 2023; Montgomery 2021). Although the Veterans Health Administration requires annual screening for alcohol use, prior research suggests that screening may not consistently capture information regarding quantity of use and occurrence of episodes of heavy drinking (Bachrach et al., 2018), and brief interventions following screening may be particularly limited among veteran women (Williams et al., 2017). Increasing screening and brief intervention efforts among justice-involved veterans, especially women, may represent an important opportunity for early intervention.

Second, problematic anger among veteran women emerged as a key area for focused support. Although previous research has found that veteran men were more likely to endorse problematic anger compared to veteran women (Adler et al., 2022), the findings of the present study suggest that problematic anger may be particularly consequential for women veterans in relation to criminal justice involvement. These findings highlight the potential value of screening for problematic anger and providing targeted interventions focused on emotion regulation among women veterans. Finally, our results underscore the need for increased attention to the experiences of younger veterans, who may be particularly vulnerable to criminal justice involvement following deployment. Younger service members may be more likely to enter the military without a college degree and may face challenges securing stable employment upon returning to civilian life. Considering that post-9/11 veterans are significantly younger than veterans from previous service eras (Waszak & Holmes 2017), many may encounter simultaneous challenges related to education, employment, and social role transitions during reintegration. Facilitating connections to educational and vocational resources through the Department of Veterans Affairs may therefore be an important strategy for supporting successful transition to civilian life and reducing risk of criminal justice involvement. However, many veterans report limited awareness of available resources, which can lead to delayed engagement with necessary services (Stryczek et al., 2023). Clinicians and other providers may play an important role in helping veterans navigate these systems by discussing available resources and facilitating connections to services, including mental health and substance use treatment, employment assistance, and housing support.

Future research should continue to examine the mechanisms linking mental health, psychosocial factors, and criminal justice involvement among veterans. This research would benefit from incorporating comprehensive, multi-item measures of justice involvement to better capture the frequency, type, and severity of legal system contact. Additionally, future research would benefit from the use of administrative records related to criminal justice involvement to reduce potential self-report bias. Longitudinal studies are particularly needed to clarify the temporal relationships among these factors and to identify critical windows for prevention and intervention. Finally, additional research is needed to examine how risk factors for criminal justice involvement may vary across demographic subgroups, including potential intersectional patterns among women veterans who are also members of racial/ethnic or sexual and gender minority groups.

## Conclusion

In summary, this study examined gender differences in correlates of post-deployment criminal justice system involvement among post-9/11 veterans. Several factors were associated with criminal justice involvement across genders, including younger age and a provisional diagnosis of alcohol use disorder, while additional gender-specific correlates emerged. Among women, problematic anger was uniquely associated with criminal justice involvement. whereas Among men, lifetime history of suicide attempts and post-deployment homelessness were positively, and a provisional diagnosis of somatoform disorder was negatively, associated with criminal justice involvement. These findings highlight the importance of considering both shared and gender-specific pathways when identifying veterans at risk for justice involvement. Efforts to reduce criminal justice system involvement among veterans may benefit from integrated approaches that address substance use, suicide risk, housing instability, and emotional regulation while supporting veterans during the transition from military to civilian life.

## Data Availability

Raw data are not publicly available to preserve individuals’ privacy per the U.S. Department of Veteran Affairs and Institutional Review Board regulations.
